# Electroclinical Mismatch During EEG Acquisition: What It Might Mean, What We Might Need to Do

**DOI:** 10.7759/cureus.23122

**Published:** 2022-03-13

**Authors:** Anil K Chimakurthy, Nicole R Villemarette-Pittman, Maxwell H Levy, Piotr W Olejniczak, Edward C Mader

**Affiliations:** 1 Neurology, Louisiana State University Health Sciences Center, New Orleans, USA; 2 Neurology, Tulane University School of Medicine, New Orleans, USA

**Keywords:** benzodiazepine challenge, lorazepam, status epilepticus, seizure, mismatch, electroclinical, encephalopathy, epileptiform, eeg

## Abstract

An electroclinical mismatch is present if the electroencephalogram (EEG) shows evidence of moderate to severe diffuse encephalopathy but the patient’s mental status is only mildly altered. We describe five cases in which seizure or status epilepticus was suspected due to electroclinical mismatch. In all five cases, EEG was ordered to rule out nonconvulsive status epilepticus as the cause of the altered mental status. EEG initially showed generalized delta activity (GDA), with variable degrees of rhythmicity, with or without superimposed theta activity, with or without sporadic epileptiform discharges. During EEG acquisition, all patients followed commands and answered questions. The mental status change was limited to mild inattention and temporal disorientation. Benzodiazepine challenge was performed by administering lorazepam 2-mg IV. Within 10 minutes of injection, GDA started to break up and subsequently disappeared. EEG showed prominent sleep spindles in three patients and background changes, indicating drowsiness in two patients. The assessment of clinical response to lorazepam was confounded by sleepiness in all patients. Serial EEG recording or continuous EEG monitoring revealed reemergence of GDA, at times appearing more rhythmic than the GDA in the baseline study. All patients received nonsedating antiseizure drugs. GDA completely resolved and mental status normalized two to five days after starting antiseizure medication. In cases of electroclinical mismatch, the absence of clear-cut epileptiform discharges does not exclude the possibility that cortical hyperexcitability is contributing to the encephalopathic process. A positive response to benzodiazepine challenge suggests the presence of cortical hyperexcitability and the need to start, or increase the dosage of, antiseizure drugs.

## Introduction

A direct correlation exists between the degree of slowing on scalp electroencephalogram (EEG), the depth of toxic-metabolic encephalopathy, and the severity of mental status change [1-2]. Sustained generalized delta activity (GDA) implies moderate to severe encephalopathy. A person with this degree of encephalopathy is expected to be comatose, stuporous, obtunded, or profoundly delirious [3]. Sometimes, we encounter a paradoxical situation wherein the EEG shows sustained GDA, but the patient’s mental status is only minimally altered, e.g., the person is only mildly inattentive or has minimal confusion. We use the term “electroclinical mismatch” to describe this phenomenon [4].

Three GDA patterns can be identified by visually inspecting the EEG: rhythmic GDA, quasirhythmic GDA, and polymorphic GDA [5]. Continuous EEG (cEEG) recording may also show fluctuations in GDA rhythmicity, e.g., GDA may be highly rhythmic at times and quasirhythmic at other times. According to the American Clinical Neurophysiology Society (ACNS) nomenclature for rhythmic and periodic EEG patterns [6], the proper terminology is generalized rhythmic delta activity (GRDA), not rhythmic GDA. However, the term rhythmic GDA is a better match for the terms quasirhythmic GDA and polymorphic GDA. Regardless of the degree of rhythmicity, GDA with an average frequency below 2.5 Hz and without definite evolutionary change does not fulfill the Salzburg consensus criteria for nonconvulsive status epilepticus [7]. Likewise, sustained rhythmic GDA that is not part of an evolving pattern does not satisfy the definition of electrographic seizure or status epilepticus and rhythmic GDA in a patient with altered mental status, as the only clinical manifestation does not satisfy the definition of electroclinical seizure or status epilepticus [6].

Benzodiazepine challenge has been utilized for decades to support the diagnosis of seizure or status epilepticus [8]. Most protocols use lorazepam 2-mg IV giving rise to the “Ativan challenge” as medical parlance. The EEG is inspected for suppression of suspected epileptiform activity, and the patient is examined for improvement in mental status. When EEG is not readily available, a clear-cut clinical response may be enough to support the diagnosis of status epilepticus [9]. Both the Salzburg criteria for nonconvulsive status epilepticus and the ACNS definitions of electrographic and electroclinical seizure or status epilepticus take benzodiazepine responsiveness into account [6-7]. The observation that benzodiazepines can suppress triphasic waves (which have been viewed for decades as the EEG hallmark of metabolic encephalopathy) led some experts to question the validity of benzodiazepine challenge for seizure diagnosis [10].

We describe five cases of electroclinical mismatch, explore the significance of this phenomenon, and discuss the usefulness and shortcomings of the current benzodiazepine challenge and mental status testing protocols during EEG acquisition.

## Case presentation

This case series includes five patients, herein designated as Patient-1 to Patient-5. All patients underwent a stat EEG because of an acute change in mental status. An electroclinical mismatch was observed in all patients during the initial EEG recording. EEG showed rhythmic or quasirhythmic GDA, with variable amounts of superimposed generalized rhythmic theta activity, with or without superimposed sporadic epileptiform discharges. Although somewhat drowsy during EEG acquisition, all patients were able to follow verbal commands and answer the questions of the technologist. Based on video and record annotations, mental status impairment was limited to mild inattention and temporal disorientation. Seizure or status epilepticus was suspected because of electroclinical mismatch. Benzodiazepine challenge (lorazepam 2-mg IV) was performed in all five patients. Within five to 10 minutes of injection, GDA started to attenuate or break up as generalized rhythmic theta activity and/or sleep spindles emerged. All patients became somnolent after receiving intravenous lorazepam, confounding the assessment of clinical response to benzodiazepine challenge. Serial EEG recording or cEEG monitoring showed the reemergence of rhythmic GDA (at times more rhythmic when compared to the baseline study) and/or the appearance of various expressions of cortical hyperexcitability. All patients received nonsedating IV antiseizure medications. GDA resolved completely and mental status normalized within two to five days.

**Figure 1:**
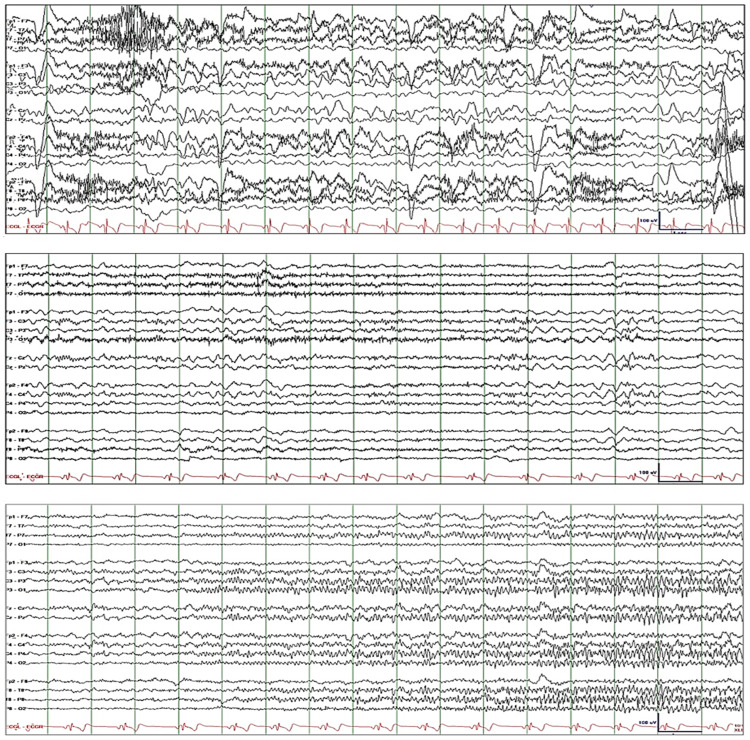
Patient-1. Top tracing: EEG initially showed quasirhythmic GDA, fluctuating in amplitude and frequency, with superimposed rhythmic theta activity. Middle tracing: Lorazepam 2-mg IV resulted in attenuation of GDA and intermittent appearance of quasirhythmic theta and rhythmic beta activity. Bottom tracing: EEG the next day showed further attenuation of GDA and evidence of cortical hyperexcitability in the right centroparietal region, including focal electrographic seizures and sporadic epileptiform spikes. EEG: electroencephalography; GDA: generalized delta activity

Patient-1 is a 40-year-old female with childhood-onset epilepsy with focal unaware seizures who presented with a two-day history of frequent breakthrough seizures. While she was in the emergency room, she had two episodes of focal unaware seizures. She received 2 mg of lorazepam five hours before EEG was recorded. The initial EEG showed quasirhythmic GDA and superimposed rhythmic theta activity fluctuating in amplitude and frequency (Figure 1: top tracing). She was drowsy but every time she was alerted, she was able to execute simple commands and she responded to questions correctly, except when asked the month and year. Lorazepam 2-mg IV resulted in attenuation of GDA and intermittent appearance of quasirhythmic theta and rhythmic beta activity (Figure 1: middle tracing). She was somnolent; when alerted, she immediately went back to sleep. EEG the next day showed further attenuation of GDA and evidence of cortical hyperexcitability in the right centroparietal region, including focal electrographic seizures and sporadic epileptiform spikes (Figure 1: bottom tracing). The patient received levetiracetam 1500-mg IV q12h, lacosamide 100-mg IV q12h, and dexamethasone 5-mg IV q8h. GDA resolved completely after four days, and mental status normalized after five days.

**Figure 2:**
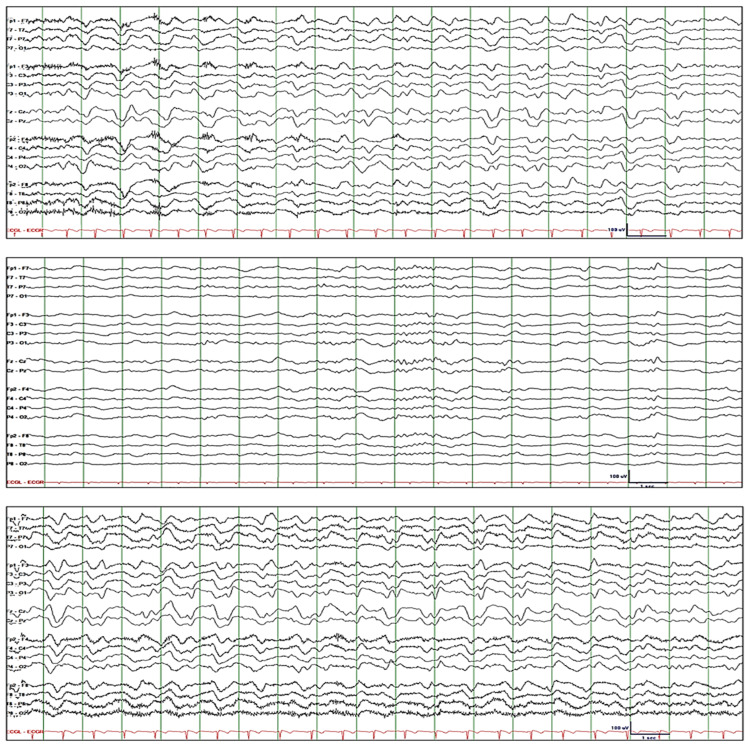
Patient-2. Top tracing: EEG initially showed 1.5 to 2.5 Hz rhythmic GDA with fluctuating amplitude. Middle tracing: Lorazepam 2-mg IV resulted in attenuation of GDA and appearance of spindle-like rhythms. Bottom tracing: cEEG showed reappearance of rhythmic GDA, which reached baseline levels approximately two hours after injection of lorazepam and persisted for about 30 hours before starting to break up and attenuate. EEG: electroencephalography; GDA: generalized delta activity

Patient-2 is a 30-year-old male who was recently diagnosed with testicular cancer. After completing a course of chemotherapy, the patient developed acute kidney injury and presented with delirium. His mental status continued to wax and wane despite correction of metabolic disturbances; hence, EEG was requested. EEG initially showed 1.5 to 2.5 Hz rhythmic GDA with fluctuating amplitude (Figure 2: top tracing). He was slightly drowsy but was easily alerted by verbal stimuli, and he responded correctly to commands and questions (except when asked the month and year). Lorazepam 2-mg IV resulted in attenuation of GDA and intermittent appearance of spindle-like rhythms (Figure 2: middle tracing). He was somnolent but, when alerted by verbal or tactile stimuli, he responded appropriately to some questions. Forty-eight hours of cEEG monitoring showed gradual reappearance of rhythmic GDA, which reached baseline levels approximately two hours after injection of lorazepam. GDA persisted for about 30 hours before starting to break up and attenuate (Figure 2: bottom tracing). The patient received levetiracetam 2000-mg IV load then 1500-mg IV q12h and valproate sodium 1000-mg IV load then 500-mg q8h. GDA resolved completely after 1.5 days, and mental status normalized after two days.

**Figure 3:**
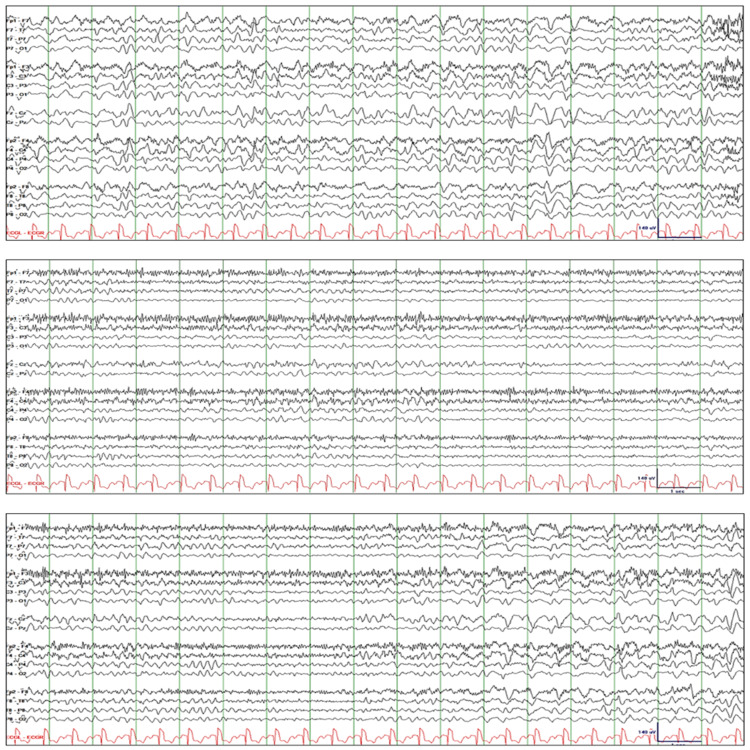
Patient-3. Top tracing: EEG initially showed quasirhythmic GDA, with fluctuating amplitude and frequency, with superimposed rhythmic theta activity. Middle tracing: Lorazepam 2 mg IV resulted in suppression of rhythmic GDA and appearance of anteriorly predominant rhythmic beta activity and generalized quasirhythmic theta activity. Bottom tracing: cEEG the next day showed generalized rhythmic theta activity with intermittent bursts of rhythmic/periodic GDA at times with triphasic morphology. Rhythmic GDA gradually disappeared and rhythmic theta activity increasingly became predominant. EEG: electroencephalography; GDA: generalized delta activity

*Patient-3 *is a 53-year-old female with diabetes mellitus and end-stage kidney disease. After three hours of hemodialysis, she experienced a focal unaware seizure with right-sided gaze deviation and moaning. The episode lasted about two minutes. Stat EEG was ordered due to prolonged postictal somnolence. The EEG demonstrated quasirhythmic GDA and superimposed rhythmic theta activity with fluctuating amplitude and frequency (Figure 3: top tracing). She was drowsy but was able to converse whenever she was alerted. She responded correctly to all commands and questions. Lorazepam 2-mg IV resulted in suppression of rhythmic GDA and appearance of anteriorly predominant rhythmic beta activity and generalized quasirhythmic theta activity (Figure 3: middle tracing). When alerted, she rapidly went back to sleep. The next day, cEEG showed generalized rhythmic theta activity with intermittent bursts of rhythmic/periodic GDA at times with triphasic morphology (Figure 3: bottom tracing). Rhythmic GDA gradually disappeared, and rhythmic theta activity increasingly became predominant. The patient received levetiracetam 1500-mg IV load then 1000-mg IV q12h and PRN lorazepam 2-mg IV x three doses over 48h. GDA resolved completely after four days, and mental status normalized after five days.

**Figure 4:**
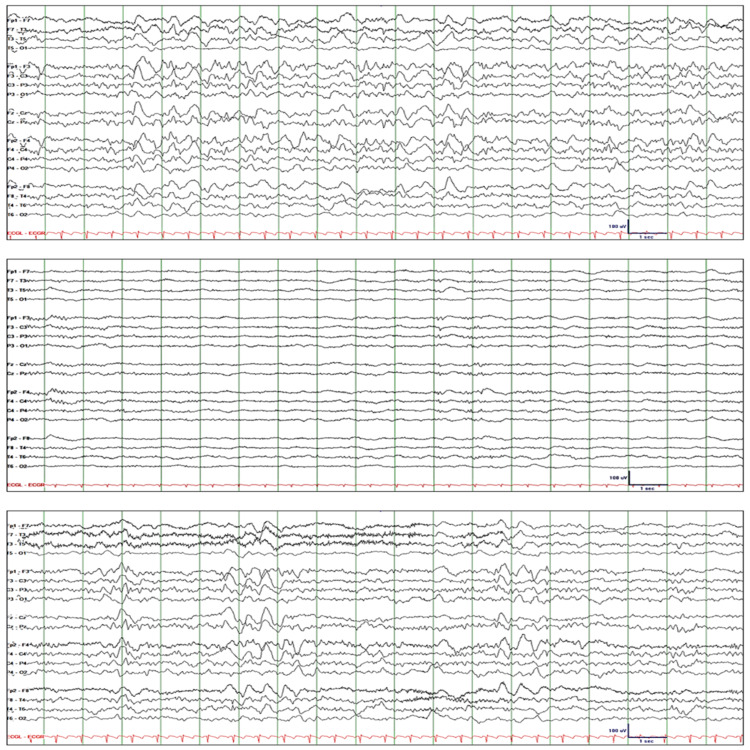
Patient-4. Top tracing: EEG initially showed waxing and waning quasirhythmic GDA with superimposed rhythmic theta and beta activity. Middle tracing: Lorazepam 2 mg IV resulted in attenuation of GDA and intermittent appearance of spindle rhythm. Bottom tracing: Repeat EEG the next day showed bursts of rhythmic GDA becoming progressively shorter and less frequent. Some bursts of rhythmic GDA are reminiscent of FIRDA. EEG: electroencephalography; GDA: generalized delta activity; FIRDA: frontal intermittent rhythmic delta activity

Patient-4 is a 31-year-old male with diabetes mellitus and opiate and sedative-use disorder who presented with depressed sensorium. The patient was found to be hypoglycemic in the emergency room. Stat EEG was ordered because mental status failed to improve immediately after IV glucose was administered. Initial EEG showed waxing and waning quasirhythmic GDA with superimposed rhythmic theta and beta activity (Figure 4: top tracing). He was somnolent but when alerted he responded correctly to commands and questions, except when asked the month and year. Lorazepam 2-mg IV resulted in attenuation of GDA and intermittent appearance of spindle rhythm. He was sleepier compared to baseline, but he was easy to alert, and he responded appropriately to questions (Figure 4: middle tracing). The next day, EEG showed the episodes of generalized rhythmic delta activity progressively becoming shorter in duration and less frequent in occurrence (Figure 4: bottom tracing). Some bursts of rhythmic GDA started to look like frontal intermittent rhythmic delta activity (FIRDA). The patient received levetiracetam 1000-mg IV load then 500-mg IV q12h. GDA and FIRDA resolved completely after two days, and mental status normalized after three days.

**Figure 5:**
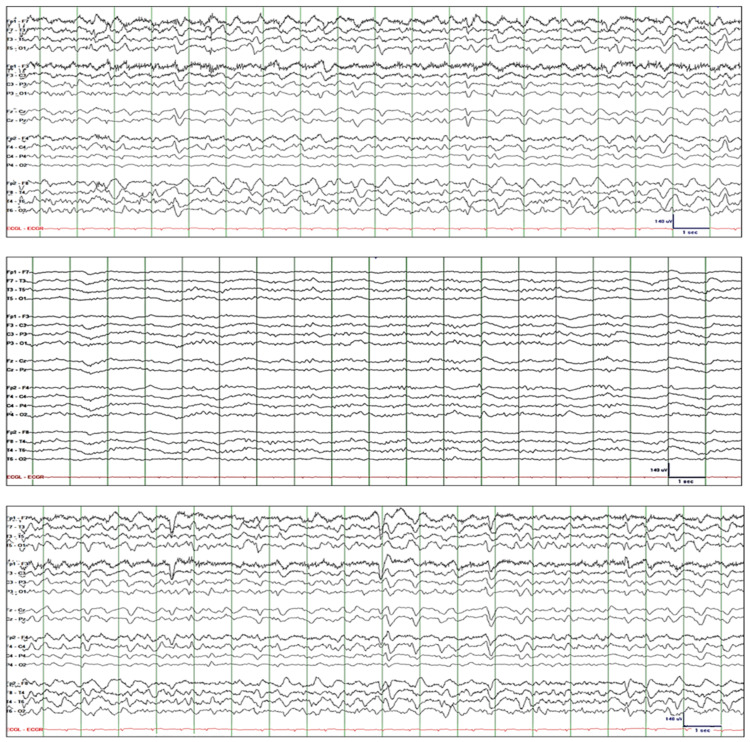
Patient-5. Top tracing: EEG initially showed 1.5 to 2.5 Hz rhythmic GDA at times with sporadic or rhythmic sharp waves. Middle tracing: Lorazepam 2 mg IV resulted in marked attenuation of rhythmic GDA and appearance of rhythmic theta activity. Bottom tracing: cEEG the next day showed rhythmic GDA similar to the baseline study, but this time with abundant sharp waves and behavioral manifestations such as staring and constant blinking indicating electroclinical status epilepticus. EEG: electroencephalography; GDA: generalized delta activity; FIRDA: frontal intermittent rhythmic delta activity

Patient-5 is a 43-year-old female who presented with confusion, hypoxia, and hypercapnia. This was attributed to suboxone, gabapentin, and quetiapine. Her past medical history was significant for a psychiatric disorder, opiate and sedative-use disorder, and interstitial lung disease. Stat EEG was ordered to rule out nonconvulsive status epilepticus. EEG initially showed 1.5 to 2.5 Hz rhythmic GDA with superimposed sharp waves (Figure 5: top tracing). She was drowsy, and she appeared in a daze when alerted. However, she responded correctly to all commands and questions. Lorazepam 2-mg IV resulted in attenuation of rhythmic GDA and appearance of rhythmic theta activity (Figure 5: middle tracing). She became very sleepy, and when aroused, she simply moaned and refused to follow commands and answer questions. The next day, cEEG showed rhythmic GDA similar to the baseline study, but this time with abundant sharp waves and behavioral manifestations such as staring and constant blinking indicating electroclinical status epilepticus (Figure 5: bottom tracing). The patient received levetiracetam 1500-mg IV q12h, lacosamide 100-mg IV q12h, valproate 500-mg IV q8h, and methylprednisolone 1000-mg IV q24h. GDA resolved completely, and mental status normalized after five days.

## Discussion

Neuronal excitability is an ever-changing physiological parameter of neurons and neural networks [11]. A net increase in cortical-subcortical network excitability (lowering of the seizure threshold) predisposes a person to seizures [12]. Cortical hyperexcitability may be expressed in the EEG as sporadic, periodic, or rhythmic epileptiform discharges, or as electrographic seizures [5-6]. In most hospitals, recording the EEG on the scalp and identifying epileptiform discharges is the only means to detect cortical hyperexcitability. In practice, the scalp-recorded EEG may not show epileptiform discharges even when cortical hyperexcitability is present. EEG professionals are well-aware that at least 6 square centimeters of cortex must discharge simultaneously before an electrical potential can be recorded on the scalp [13]. In patients with temporal lobe epilepsy, only 10% of cortical spikes with a source area of less than 10 square centimeters are associated with a spike on the scalp [14]. Decades of experience with invasive EEG recording employing subdural and other intracranial electrodes in epilepsy surgery candidates has increased our awareness of the limitations of the scalp EEG for studying interictal and ictal epileptic activity [15-17].

Cortical hyperexcitability can escape diagnosis when scalp EEG waves are not clearly epileptiform such as slow waves with no spike components and rhythmic slow waves with a frequency less than 2.5 Hz [6-7]. By itself, a slow wave is not epileptiform, but a slow wave trailing a spike is an integral part of the epileptiform discharge or spike-and-wave complex [18]. The spike represents cortical activation, and the slow wave represents surround inhibition, the latter serving as a deterrent to the spread of cortical excitation [19]. Slow waves with no sharp components can still be a harbinger of cortical hyperexcitability. Scalp-recorded temporal intermittent rhythmic delta activity implies temporal lobe hyperexcitability [20]. Interictal regional delta slowing recorded with electrocorticography is a marker of the epileptic network in temporal lobe epilepsy [21]. Temporal lobe seizures are frequently accompanied by frontoparietal slow-wave activity, the latter contributing substantially to ictal semiology [22]. A recent combined ictal PET-EEG study revealed cortical hypermetabolism (the PET signature of cortical hyperexcitability) concurrent with scalp-recorded focal rhythmic delta activity [23]. Most generalized seizures are expressed on the EEG as spike and slow-wave discharges. Interestingly, the ictal correlate of absence seizure in some children with absence epilepsy is rhythmic GDA, not generalized spike and wave discharges [24-25].

Toxic-metabolic encephalopathy is expressed electrographically as generalized theta, mixed theta, and delta, or predominantly delta activity (i.e., GDA) with variable rhythmicity. Although EEG background slowing is the hallmark of diffuse encephalopathy, it is not uncommon to find EEG signs of cortical hyperexcitability, such as multifocal sharp waves or electrographic seizures, in patients with toxic-metabolic encephalopathy [26]. In fact, the risk of encephalopathy and epileptic activity are both enhanced by uremic neurotoxins [27] and by the neurotoxic drug, cefepime [28]. It can be hard to determine if an EEG pattern represents encephalopathy, cortical hyperexcitability, or both. A classic example is triphasic waves, a pattern once thought to be the hallmark of hepatic encephalopathy but now considered an expression of cortical hyperexcitability, particularly nonconvulsive status epilepticus [29]. Indeed, a spectrum of EEG abnormalities may be present in toxic-metabolic encephalopathy with slow-wave activity at one end, slow-wave activity and sporadic epileptiform discharges in the middle, and full-blown seizures or status epilepticus at the other end [26]. Drawing a line between what is encephalopathic and what is epileptic can be difficult, not only because of the overlap between encephalopathy and cortical hyperexcitability but also because both toxic-metabolic encephalopathy and status epilepticus are dynamic processes with EEG findings that change from time to time [1,30].

The five cases described in this paper are similar to the three cases reported by Uthman et al. [31]. GDA implies moderate to severe encephalopathy but the patients only showed mild alteration in mental status. On the grounds of electroclinical mismatch, the history of seizures (all three cases of Uthman et al. and four of the five cases we presented had seizures), the subsequent emergence of epileptiform discharges, and the response to antiseizure drugs, Uthman et al. argued that GDA can be an unusual EEG correlate of nonconvulsive status epilepticus [31]. A multicenter study of periodic and rhythmic EEG patterns in critically ill patients concluded that GRDA (i.e., rhythmic GDA) is not associated with an increased risk of seizures [32]. However, a lack of association between an EEG pattern and seizure risk does not exclude the possibility that the pattern indicates thalamocortical hyperexcitability and hypersynchrony. Indeed, widespread thalamocortical oscillatory activity may have a role in terminating seizures and deterring cortical ictogenesis [33]. Moreover, as mentioned earlier, there is an overlap between encephalopathy and status epilepticus and, in both conditions, EEG findings may fluctuate with time [26,30]. In any case, whether or not GDA is a direct correlate of status epilepticus, the possibility that some form of cortical hyperexcitability is contributing to the encephalopathic process must be considered whenever electroclinical mismatch is present.

The benzodiazepine challenge (lorazepam 2-mg IV) was performed, and a clear-cut electrographic response was observed in all of our patients: GDA started to break up and subsequently disappeared within 10 minutes of injection. EEG showed prominent sleep spindles in three patients, and background changes indicating drowsiness in two patients. Unfortunately, demonstrating a clinical response was difficult because of the mild baseline deficits, the lack of detailed cognitive assessment (the EEG technologist performed the same routine protocol), and the emergence of sleep after lorazepam injection. Serial EEG recording or cEEG monitoring eventually showed a reemergence of GDA, at times more rhythmic when compared to the GDA in the baseline study. All patients received nonsedating antiseizure drugs. GDA completely resolved and mental status normalized two to five days after starting antiseizure medication.

A limitation of this observational study is the occurrence of sleepiness after lorazepam 2-mg IV, which prevented a proper assessment of clinical response. A smaller dose of lorazepam (e.g., 1-mg IV) should have been tried first. Another limitation is the reliance on the technologist annotations and recorded video to assess the mental status. Mental status should be assessed using a standard instrument such as the Confusion Assessment Method Scale for ICU Delirium (CAM-ICU) [34]. The CAM-ICU has been validated for use in mechanically ventilated patients with altered mental status or status epilepticus. Future research using standardized tools and better benzodiazepine challenge protocols is needed to understand the phenomenon of electroclinical mismatch.

## Conclusions

Generalized delta activity (GDA), the EEG hallmark of moderate to severe encephalopathy, is typically seen in patients who are stuporous, obtunded, or profoundly confused. A person with GDA who is only mildly inattentive presents a paradox, known as an electroclinical mismatch. The electroclinical mismatch suggests the possibility that cortical hyperexcitability is contributing to the pathophysiology of GDA and mental status change. Since GDA with frequency <2.5 Hz does not meet the electrographic criteria for seizure or status epilepticus, the physician should consider performing a benzodiazepine challenge with a smaller dose of IV lorazepam (e.g. 1-mg) since the usual dose (2-mg) can induce somnolence and confound clinical response assessment. Although a positive response is only supportive (not confirmatory) of abnormal cortical hyperexcitability, it justifies treating with (or increasing the dose) of nonsedating antiseizure drugs. Serial or continuous EEG recording may reveal epileptiform discharges and guide treatment decisions down the road. Future studies are needed to validate this empiric approach to electroclinical mismatch and to reach an expert consensus on the optimal method for mental status assessment during EEG acquisition.
